# Fixed-Bed Adsorption: Comparisons of Virgin and Zirconium Oxide-Coated Scoria for the Removal of Fluoride from Water

**DOI:** 10.3390/molecules27082527

**Published:** 2022-04-14

**Authors:** Wondwosen Sime Geleta, Esayas Alemayehu, Bernd Lennartz

**Affiliations:** 1 School of Chemical Engineering, Jimma Institute of Technology, Jimma University, Jimma P.O. Box 378, Ethiopia; 2 Faculty of Agricultural and Environmental Sciences, University of Rostock, Justus-Von-Liebig-Weg 6, 18059 Rostock, Germany; 3 Faculty of Civil and Environmental Engineering, Jimma Institute of Technology, Jimma University, Jimma P.O. Box 378, Ethiopia; 4 Africa Center of Excellence for Water Management, Addis Ababa University, Addis Ababa P.O. Box 1176, Ethiopia

**Keywords:** adsorption, fluoride, virgin scoria, zirconium oxide-coated scoria

## Abstract

Many people worldwide are exposed to extreme levels of fluoride in drinking water. It is, therefore, critical to develop inexpensive, locally available, and environmentally friendly adsorbents for fluoride-laden water defluoridation. In the current study, virgin scoria (volcanic rock) from Ethiopia, was modified with zirconium oxide and used as an adsorbent in a fixed-bed column aiming at the removal of fluoride from water. The adsorption capability of zirconium oxide-coated scoria (ZrOCSc) was compared with unmodified virgin scoria (VSco). XRD, FTIR, XRF, SEM, ICP-OES, and the pH_PZC_ tests were evaluated to explore the adsorption mechanisms. Thermal analysis of VSco and ZrOCSc revealed lower total weight losses of 2.3 and 3.2 percent, respectively, owing to the removal of water molecules and OH species linked to metal oxides contained in the material. The effect of test conditions such as the pH of the solution and the influent flow rate on the adsorption capacity of the adsorbent was carefully studied. ZrOCSc exhibited the maximum removal capacity of 58 mg/kg, which was 4.46 times higher than the observations for VSco (13 mg/kg) at pH 2, and an initial flow rate of 1.25 mL/min. Breakthrough time increased with decreasing initial pH and flow rate. The adsorption experimental data under various test conditions were examined by the Thomas and Adams–Bohart models. Both models were found very effective in describing the experimental data with a correlation coefficient (R^2^) of ≥0.976 (ZrOCSc) and ≥0.967 (VSco). Generally, coating VSco with zirconium oxide improved the adsorption performance of VSco; hence, a ZrOCSc-packed fixed bed could be employed for the decontamination of high levels of fluoride from groundwater. However, further examination of the adsorbent using natural groundwater is advisable to produce a definitive conclusion.

## 1. Introduction

Fluorine is the thirteenth most abundant element and makes up between 0.06% and 0.09% of the entire Earth’s crust [1,2]. Fluorine is always in a combined form of minerals such as fluoride. Fluoride levels in surface water ranged from 0.01 to 0.3 mg/L, while groundwater levels range from less than 1 to more than 35 mg/L [2,3]. Fluoride is thought to have beneficial effects in trace amounts in drinking water, but prolonged exposure to fluoride in drinking water, or combined effect with exposure to fluoride from other sources, could result in some negative effects [4,5,6,7]. It can prevent the incidence of dental caries, particularly in children under the age of 8 years, if taken in the drinking water at an optimum level (~0.5–1.5 mg/L) [8]. However, if the permissible level is exceeded, dental fluorosis or mottled enamel will appear, and if the concentration is greater than 3 mg/L, it will also cause skeletal fluorosis [9]. 

Groundwater is the safest and most economically viable option of all available drinking water sources for many communities around the world, as is the case for many communities in rural and urban areas of the main African rift valley [4,5,7]. In recent years, the entry of geogenic pollutants, such as fluoride, into groundwater aquifers has become a serious environmental problem worldwide. Over 200 million people around the world, including in East Africa, are drinking fluoride-containing groundwater beyond the permitted limit (1.5 mg/L), which has a significant impact on human well-being [5,7,10]. Fluorosis is by far the most common geochemical disease in the East African rift, impacting more than 80 million individuals [7,11,12]. Ethiopia is one of the most populous East African countries where excessive fluoride is becoming a growing issue, particularly along the main rift of the country [13,14]. The fluoride concentrations in Ethiopian rift wells are usually 1 to 10 times higher than the WHO standard, which puts about 10 million Ethiopians at high risk of fluoride ion exposure [13,15]. 

High geogenic fluoride levels in groundwater are related to various geological climatic conditions such as arid climates, granitic basements, and alkaline volcanic rocks. In addition to the natural geological sources for fluoride enrichment in groundwater, numerous fluorochemical industries including aluminum smelting are also contributors to fluoride contamination. Alkaline volcanic areas, such as the East African Rift Valley, have some of the highest fluoride concentrations since high-fluoride hyper-alkaline volcanic rocks are present and fluoride is also introduced to groundwater through high-fluoride geothermal solutions [16,17]. Low calcium levels can also cause too high fluoride levels in groundwater. The weathering of primary rocks and the leaching of fluoride-containing minerals in the soil exacerbate the problem of excessive fluoride in groundwater in the Ethiopian Rift, which is generally linked with a low calcium content and high concentrations of bicarbonate [18,19]. Therefore, a high concentration of fluoride in groundwater is among the most pressing problems that need to be addressed urgently.

Different treatment techniques such as ion exchange, membrane, precipitation, and adsorption have been employed for the uptake of fluoride [20]. Among the existing techniques, adsorption remains the most widely employed and most suitable method because of its applicability for uptake of fluoride even at small doses, economic feasibility, high efficiency, and simplicity of design [5,7,20]. 

The adsorbent materials researched for fluoride uptake are abundant [21,22,23,24,25,26]. Nevertheless, many of them are suffering from either a time-taking synthesis procedure, high costs of processing, inaccessibility of raw materials, or short shelf life, which makes them unrealistic for use in the rift valleys’ water [5,7,27]. Thus, for sustainable defluoridation of drinking water, the search for suitable fluoride adsorbents is a critical concern for developing countries such as Ethiopia. Modifying the physicochemical properties of locally available adsorbents is also of interest, as it could have a potential for further cost reduction and applicability [5]. 

Volcanic rock (scoria) is one such indigenous material in many nations including Ethiopia that could be used as a raw material for producing an adsorbent for fluoride removal. Scoria has valuable features such as low cost, easy access, good mechanical strength, and availability in considerable quantities [7,28,29]. The possession of good mechanical strength could enable scoria to prevail over drawbacks such as clogging and/or low hydraulic conductivities in fixed-bed column adsorption techniques. However, the fluoride uptake capacity of natural scoria is limited, and surface modifications for improved performance appear not to have been well studied. Surface modification of natural adsorbents that may contribute to the available active sites for fluoride adsorption is expected to have good reactivity/affinity for fluoride ions [5,30]. From a previous study, it was generally noticed that natural materials modified with multivalent metal cations such as Mn^4+^, Zr^4+^, and Fe^3+^ can change the surface properties and the affinity of fluoride [5,31]. Among these, zirconium (Zr^4+^) is receiving more attention because of its high binding affinity to fluoride ions, non-toxicity, and acceptable cost [5,32,33]. Therefore, the study of zirconium-based adsorbents with good performance is very important. 

Taking the aforementioned problems into account, fixed-bed columns packed with zirconium (IV) oxychloride octahydrate (ZrOCl_2_·8H_2_O)-coated scoria, hereinafter abbreviated as ZrOCSc, have not been tested for the treatment of fluoride-polluted water. Therefore, the aims of the current study were to (i) compare the adsorption properties of ZrOCSc with VSco, (ii) evaluate the processes of fluoride adsorption through variations in the solution pH and influent volumetric flow rate, and (iii) describe and analyze the adsorption processes using well-known fixed-bed adsorption models such as the Thomas and Adams–Bohart model.

## 2. Results and Discussions

### 2.1. Characterization of Adsorbents

**Figure 1:**
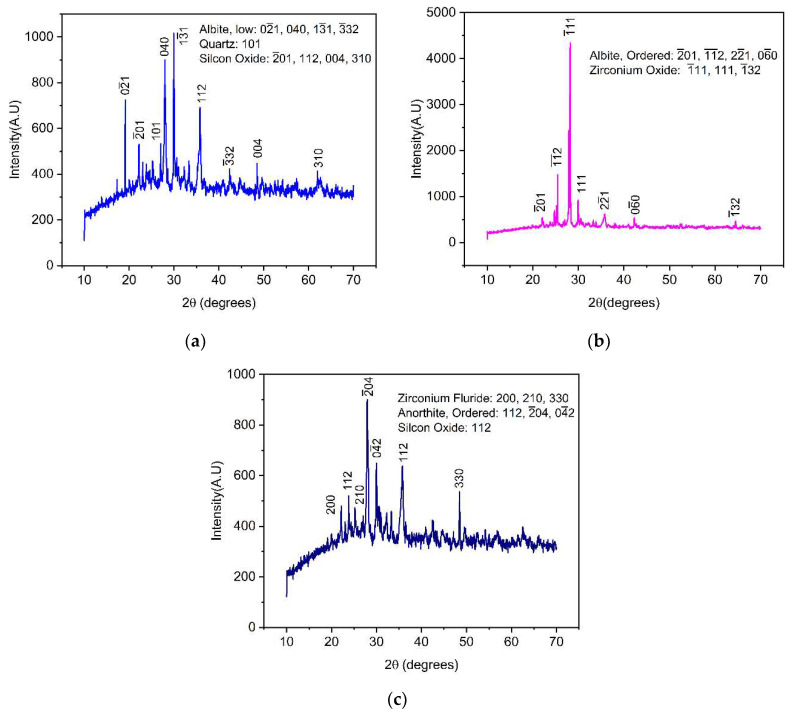
XRD patterns for (**a**) virgin scoria (VSco); zirconium oxide-coated scoria (ZrOCSc) (**b**) before and (**c**) after adsorption experiment.

The crystalline phases of VSco (before adsorption) and ZrOCSc before and after the fluoride scavenger were characterized using the XRD instrumental technique. The results show that the main crystalline phases in VSco were quartz, syn (SiO_2_) (ICSD, reference code: 01-083-2470), silicon oxide (SiO_2_) (ICSD, reference code: 01-082-1554), and albite low (Na(AlSi_3_O_8_)) (ICSD, reference code: 01-076-1819); zirconium oxide (ZrO_2_) with monoclinic crystal structure (ICSD, reference code: 01-078-0047) and albite ordered (NaAlSi_3_O_8_) (ICSD, reference code: 00-009-0466) are the main crystalline phases in ZrOCSc before adsorption, while anorthite, ordered (CaAl_2_Si_2_O_8_) (ICSD, reference code: 00-041-1486), quartz (SiO_2_) (ICSD, reference code: 01-078-0047), and zirconium fluoride (ZrF_4_) (ICSD, reference code: 01-076-1023) are the dominant components of ZrOCSc after adsorption. The prominent XRD peaks for virgin scoria (VSco) were found at 2θ = 19.10°, 22.14°, 27.01°, 27.95°, 29.91°, 35.75°, 42.35°, 48.48°, and 61.96° (Figure 1a). 

The diffraction pattern of ZrOCSc before adsorption (Figure 1b) showed prominent peaks at 2θ = 22.13°, 25.46°, 28.19°, 29.90°, 35.78°, 42.25°, and 64.49°; while the peaks at 2θ = 22.13°, 23.81°, 25.25°, 27.90°, 29.95°, 35.75°, and 48.44° were observed for ZrOCSc after adsorption (Figure 1c). The number and intensity of peaks in VSco were different from those of ZrOCSc before fluoride adsorption. The difference in the number and intensity of the peaks might be because the zirconium oxide particles had grown on the surface [5,32]. As can be observed from the XRD patterns (Figure 1), the VSco (Figure 1a) had nine peaks with different intensities, with a major peak at 2θ = 29.90° having the highest intensity, which corresponded to Na(AlSi_3_O_8_). ZrOCSc before fluoride adsorption (Figure 1b) had seven prominent peaks and the major one at 2θ = 28.19° having the highest intensity, which corresponded to ZrO_2_ formed on the surface; while ZrOCSc after adsorption (Figure 1c) had seven peaks and a major peak at 27.90° (2θ), which corresponded to CaAl_2_Si_2_O_8_. The reduction in the number of peaks on coating might be due to the growth of zirconium oxide over the surface of VSco [5,32]. The number and intensities of the peaks decreased in spent adsorbent (Figure 1c), which could be due to an increase in the amorphous nature through surface co-precipitation during the adsorption of fluoride ions [34,35] or due to a slight change in the adsorbent structural framework [5].

**Figure 2:**
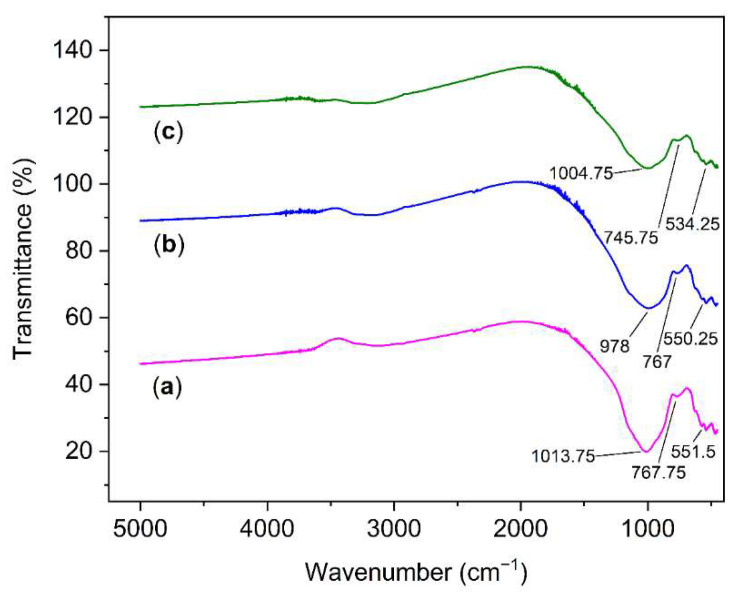
Fourier-transform infrared (FT-IR) for (**a**) VSco and for ZrOCSc (**b**) before and (**c**) after adsorption experiment.

The FTIR spectra of VSco and ZrOCSc before and after fluoride adsorption at wavelengths between 400 cm^−^^1^ and 5000 cm^−1^ are presented in Figure 2a–c, respectively. The band located at ~1011.5 and ~978 cm^−1^ can belong to the asymmetric stretching vibration of T-O-Si, T = Si or Al [5,7,36]. The peaks at ~781 and ~695.25 belong to bending vibrations of the Si-O-Si bond [5,7,37], while the band at ~767 cm^−1^ is related to the stretching vibration of 6-fold coordinated Al(VI)-OH and 6-fold coordinated Al(VI)-O [38]. The small peaks shown at ~550 cm^−1^ can be attributed to the symmetric stretching of Si-O-Si and Al-O-Si [36,39]. Although a clear diffraction peak for zirconium oxide and zirconium fluoride was detected in the XRD analysis of ZrOCSc before and after adsorption, respectively, the expected bonds—i.e., a zirconium bridge to another zirconium atom via fluorine or oxygen bridges—were not detected in the FTIR spectrum. This could be due to the FTIR equipment’s inability to detect such bonds. The inability of FTIR to detect Zr-F and F-Zr-F bending vibrations that occur at ~375–475 cm^−1^ and 375–475 cm^−1^, respectively [40], was reported for hydrous zirconium oxide after fluoride adsorption [41]. This finding is also comparable to previously reported studies [5,42,43]. However, additional investigation using complementary characterization techniques such as NMR/XPS should be considered in future work to draw strong evidence.

The chemical analysis showed that aside from the major elements in VSco were Si, Al, and Fe, as determined by ICP-OES (Table 1); Ca is the next high elemental component. Other elements were available in limited quantities or were below the device’s detection limit. The main components of VSco as measured by XRF were the oxides of Si, Fe, Ca, and Al. An earlier study reported comparable values for VSco [28]. The lack of harmful components in the VSco suggested that ZrOCSc could be useful in treating excess fluoride-laden water. The average amount of zirconium oxide coated on VSco was 1.2% (wt), while the XRF measurements showed that 8.3% (wt) zirconium oxide was coated on VSco, enhancing its fluoride removal performance. This is in line with our recent study [5].

The pH in water and the pH_PZC_ of VSco were found to be 9.3 and 8.7, respectively. These pH values are very close to the previous study [7]. The pH in water and pH_PZC_ for ZrOCSc was identified as 7.4 and 8.3, respectively. In the current study, both the pH in water and the pH_PZC_ of ZrOCSc were found to be lower than those of VSco. A similar observation was reported for zirconium-coated pumice (Zr–Pu) [5]. The surface charge of the adsorbent is positive when the pH of the solution is below pH_PZC_ ((ZrOCSc (8.3), VSco (8.7)). Thus, fluoride could be adsorbed onto the surface of adsorbents via coulombic attraction if the pH is less than pH_PZC_ [5,7].

**Figure 3:**
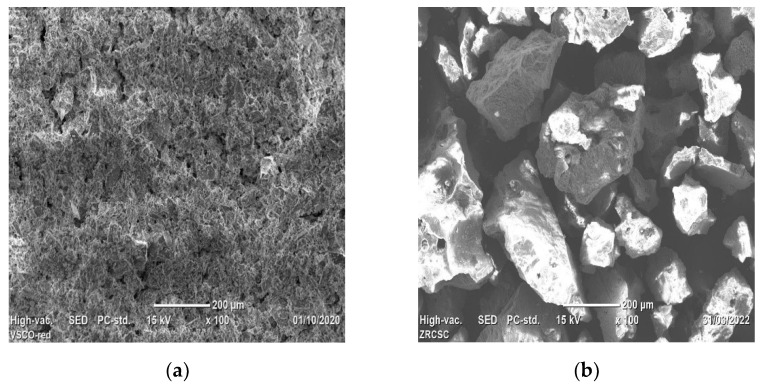
SEM image of (**a**) VSco and (**b**) ZrOCSc before adsorption experiments.

The SEM images of VSco and ZrOCSc before fluoride adsorption are shown in Figure 3a,b, respectively.

From Figure 3b, it can be seen that a large amount of irregularly shaped zirconium oxide (large clusters) is coated on the adsorbent surface and changes the surface structure of VSco. In our recent work, improvements in surface structure and adsorption for natural pumice surface modification (VPum) were reported [5].

**Figure 4:**
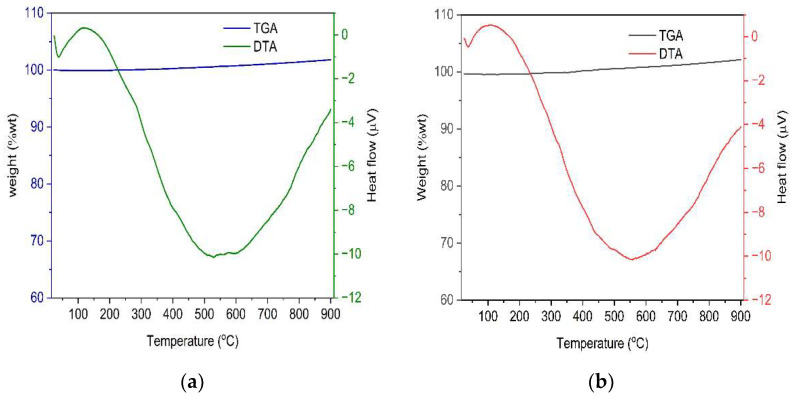
TGA-DTA thermograms of (**a**) VSco and (**b**) ZrOCSc before adsorption experiments.

Figure 4 depicts the thermal behavior of VSco (Figure 4a) and ZrOCSc (Figure 4b) at a heating rate of 15 °C/min from 25 to 900 °C under a nitrogen gas flow rate of 50 mL/min. The absorbable weight loss of about 2.3% of VSco (Figure 4a) between about 24 and 306 °C and 3.2% of ZrOCSc (Figure 4b) between 24 and 382 °C, is due to the removal of moistures as well as that of OH species linked to metal oxides and volatile organic impurities present at low levels. This demonstrates that the adsorbents could maintain their thermal stability up to 900 °C with insignificant weight gain, which could be attributed to the oxidation or reaction of the materials with nitrogen gas. An analogous observation was made in previous research [44]. The DTA thermogram of VSco (Figure 4a) showed a narrow and a broad exothermic peak in the ranges of about 25–73 °C and 204–900 °C, respectively, and an endothermic peak in the range of about 36–204 °C. A narrow and a broad exothermic peak in the region of about 25–52 °C and 185–900 °C, respectively, and an endothermic peak in the range of about 37–185 °C were also observed in the DTA analysis of ZrOCSc (Figure 4b).

### 2.2. Influence of Experimental Parameters on Fluoride Removal

#### 2.2.1. Influence of Initial Solution pH

**Figure 5:**
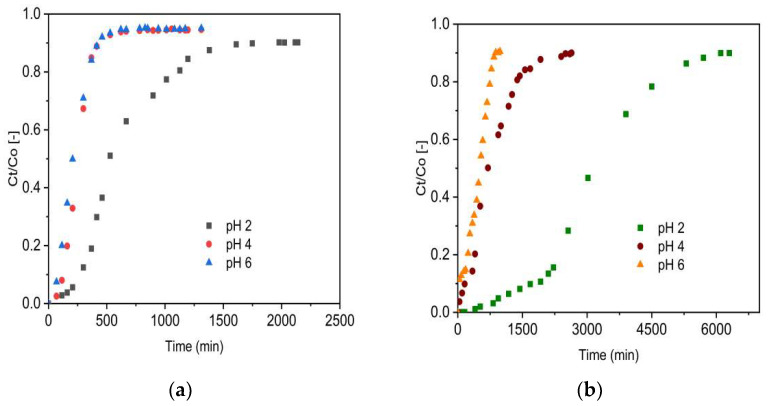
Effect of solution pH on fluoride breakthrough (**a**) VSco and (**b**) ZrOCSc (initial fluoride concentration 10 mg/L (C_O_: 10 mg/L); initial flow rate 1.25 mL/min (Q_O_: 1.25 mL/min); bed depth 10 cm).

The pH-dependent disparity in the fluoride uptake performance of the adsorbents was evaluated at various pH values (2, 4, and 6) using a separate set of fixed-bed adsorption columns. The breakthrough curves are presented for VSco (Figure 5a) and ZrOCSc (Figure 5b) for a constant inlet flow rate (1.25 mL/min), initial fluoride concentration (10 mg/L), and column bed depth (10 cm). For both adsorbents, the breakthrough curves (Figure 5) appeared to move from right to left as the pH increased from 2 to 6.

The column adsorption parameters obtained for adsorption of fluoride onto ZrOCSc and VSco are depicted in Table 2. Enhanced column performance was noticed at a lower initial solution pH, including a higher volume of treated water, enhanced defluoridation efficacy, and enhanced adsorption capacity at breakthrough and exhaustion time. The highest defluoridation capacity of 58 mg/kg and 13 mg/kg was attained for ZrOCSc and VSco, respectively. This revealed ZrOCSc scavenged 4.46 times fluoride compared with VSco. At a pH value of 2, the breakthrough capacity of 35 mg/kg (7 times that of VSco (5 mg/kg)) was achieved for ZrOCSc. A breakthrough time of 2058 min for ZrOCSc and 309 min for VSco and an exhaustion time of 3425 min for ZrOCSc and 753 min for VSco were also acquired at a pH of 2.

Thus, VSco has the briefest breakthrough and exhaustion time, whereas ZrOCSc has the longest breakthrough and exhaustion time and, hence, improved adsorption performance. When the initial pH of the solution is greater than 2, the fluoride removal rate for ZrOCSc and VSco decreases (Table 2). This may be assigned to the decrease in the amount of H^+^ or HF adsorption due to electrostatic attraction [5,7,45]. Low pH promotes protonation of the adsorbent’s surface. Increased protonation results in a greater number of positively charged sites per unit of surface area. Hence, the better adsorption performance at a pH of 2 might be corresponded to the adsorbent surface having more positive charges at lower pH and electrostatically adsorbing fluoride ions [45]. As a result, the fluoride ion sorption is caused by an electrostatic phenomenon and surface complexation, which can occur independently or in conjunction with the fluoride ion’s adsorption on the adsorbents. Furthermore, the pH value at the point of zero charges (pH_PZC_) of the adsorbents can be used to deduce this reality. The removal mechanism at pH < pH_PZC_ is possibly due to columbic attraction of fluoride by positive surface charges (Equation (1)) and/or ligand exchange reactions of fluoride with surface hydroxyl groups (Equation (2)).

(1) $${\mathrm{MOH}}_{2}^{+}+F^{-}↔{\mathrm{MOH}}_{2}^{+}---F^{-}$$

(2) $${\mathrm{MOH}}_{2}^{+}+F^{-}↔\mathrm{MF}+H_{2}O$$

where, M represents Zr, Fe, Al, Si, Ca, etc.

In general, ZrOCSc and VSco depicted similar pH-dependent defluoridation performance. Nevertheless, the zirconium oxide coating significantly improved performance owing to the specific interaction between fluoride ions and the coated zirconium (hydr) oxide. A similar observation was witnessed in a recent study [5]. At a pH of 2, the occurrence of the breakthrough time was longer, and the volume of treated water was large. As a result, the pH of the solution was kept constant at a pH of 2 in the next experiment.

#### 2.2.2. Influence of Initial Flow Rate

**Figure 6:**
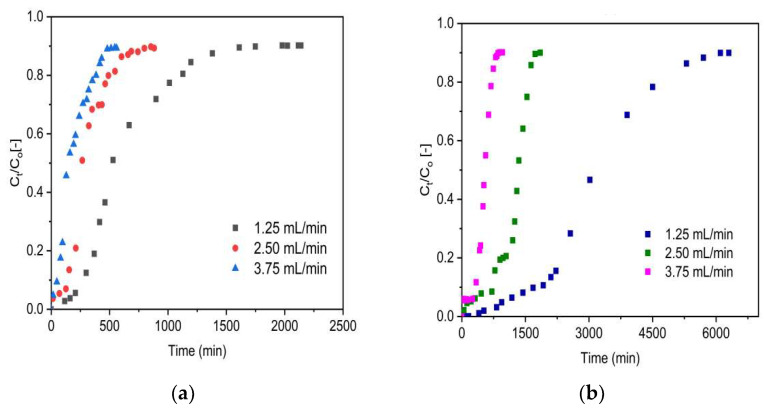
Effect of initial flow rate on the fluoride breakthrough (**a**) VSco and (**b**) ZrOCSc (pH 2; C_O_: 10 mg/L; bed depth 10 cm).

Figure 6 depicts the breakthrough curves of VSco (Figure 6a) and ZrOCSc (Figure 6b) at various flow rates (1.25, 2.50, and 3.75 mL/min), and Table 2 displays their analysis results. As anticipated, as the flow rate increases, the breakthrough time is shortened, and the breakthrough curves become steeper. This means that the fluoride solution left the column before equilibrium was reached, resulting in limited fluoride ion removal. This characteristic is due to the short residence time in the column and is also ascribed to the mass transfer zone (MTZ) (Table 2), which increases with increasing flow rate but narrows the fractional bed used [5,46]. In addition, the breakthrough and exhaustion times were lowered by increasing the flow rate (Table 2). Accordingly, the breakthrough time observed was 2058, 721, and 336 min for ZrOCSc and 309, 155, and 65 min for VSco, corresponding to the volumetric flow rates of 1.25, 2.50, and 3.75 mL/min, respectively, and the exhaustion times were 3425, 1262, and 548 min for ZrOCSc and 753, 353, and 217 min for VSco. At the breakthrough time, the amount of fluoride adsorbed (at constant initial concentration: 10 mg/L, pH: 2, and bed height: 10 cm) corresponded to 35, 24, and 17 mg/kg for ZrOCSc and 6, 5, and 3 mg/kg for VSco. The amounts adsorbed at exhaustion were 43, 32, and 21 mg/kg for ZrOCSc and 10, 9, and 8 mg/kg for VSco. This can be illustrated by the fact that as the flow rate increased, an additional amount of fluoride in the solution was exposed to adsorbents. On the other hand, the decrease in the flow rates from 3.75 to 1.25 mL/min had a significant impact on the adsorption capacity at equilibrium, so that removal capacity increased from 27.83 to 58 mg/kg for ZrOCSc and 11.02 to 12.76 mg/kg for VSco. This may be ascribed to the fact that the fluoride ions have sufficient time to diffuse through the pores of the adsorbents and hence occupy more sites at a lower flow rate. The observations coincide with different studies [5,7,47]. As the flow rate rose from 1.25 to 3.75 mL/min, the equilibrium adsorption capacity decreased, resulting in a decline in the residence time of the liquid. The empty bed contact time (EBCT) decreases due to the rise in the flow rate (Table 2), which means that at a lower flow rate, the interaction time between the adsorbent and the solution phase is longer than the larger flow rate. Our results are compatible with the findings of previous work [5,7,48].

Overall, the sensitivity of the adsorption to the flow rate of the solution can be related to the critical residence time of the solution in the column for each adsorption process. This means that the contact between the adsorbate solution and the adsorbents improved at a lower flow rate, leading to greater diffusion of fluoride ions onto the adsorbents bed and thus leading to maximum utilization of the sorption bed area. 

This influence is also evidenced in improved bed performance and hence high fluoride uptake. Even though the influence of the initial flow rate was similar for both ZrOCSc and VSco, the zirconium oxide coating showed significant improvement in fluoride adsorption capacity. The flow rate of 1.25 mL/min was observed to be optimal in this study, with a maximum fluoride uptake capacity of 58 mg/kg for ZrOCSc and 13 mg/kg for VSco, and was used in subsequent experiments.

In general, the variation in column parameters, such as q_e_, q_b_, V_e_, and V_b_, acquired for fluoride removal onto ZrOCSc and VSco under different experimental conditions demonstrated that ZrOCSc has higher activity than VSco towards fluoride (Table 2). The improved activity and thus adsorption performance could be ascribed to the coating of VSco with zirconium oxide. 

### 2.3. Application of the Thomas Model 

**Figure 7:**
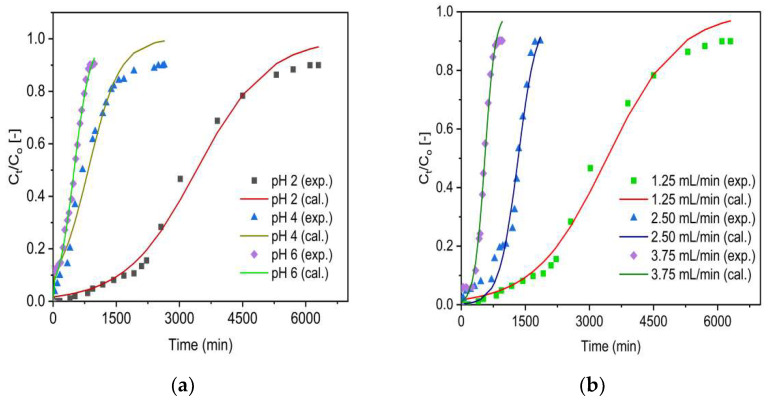
Experimental (exp.) and simulated (cal.; Thomas model) breakthrough curves of fluoride for ZrOCSc at different (**a**) pH (C_O_: 10 mg/L; Q_O_: 1.25 mL/min; bed depth 10 cm) and (**b**) initial flow rate, Q_O_ (pH 2; C_O_: 10 mg/L; bed depth 10 cm).

**Figure 8:**
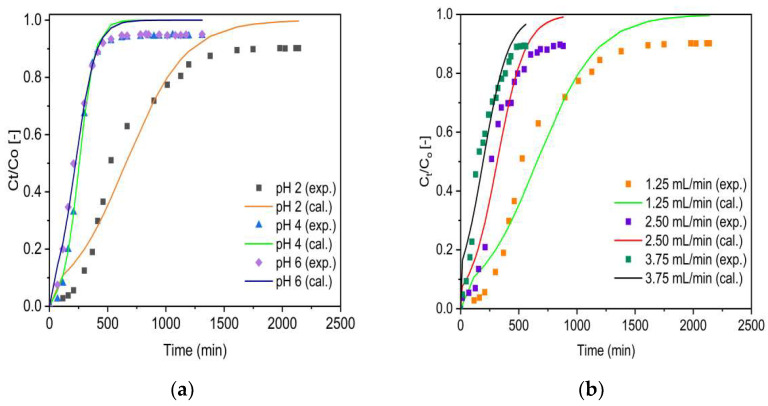
Experimental (exp.) and simulated (cal.; Thomas model) breakthrough curves of fluoride for VSco at different (**a**) pH (C_O_: 10 mg/L; Q_O_: 1.25 mL/min; bed depth 10 cm) and (**b**) initial flow rate, Q_O_ (pH 2; C_O_: 10 mg/L; bed depth 10 cm).

For evaluation of the rate constant (K_T_) and the maximum adsorption capacity (q_o_), the experimental data (denoted as exp.) and simulated data (denoted as cal.) were fitted with the non-linear Thomas model (Equation (12)). The analysis of the experimental findings related to various pH (Figure 7a,b) and flow rates (Figure 8a,b) performed on the Thomas model was predicted.

The model parameters are depicted in Table 3. As can be noted from Figure 7 and Figure 8, the breakthrough curves appeared to move from right to the left as the pH and inlet flow rate increased from 2 to 6 and 1.25 to 3.75 mL/min, respectively. For both adsorbents (ZrOCSc and VSco), the concentration values (q_o_) calculated by the Thomas model (Equation (12)) were comparable with the obtained experimental values (q_e_) (Table 3). The value of q_o_ decreased from 58 to 9 (mg/kg) for ZrOCSc and from 11 to 4 (mg/kg) for VSco with increased pH (2 to 6) and flow rates (1.25 to 3.75 mL). A higher value of K_T_ indicated a faster approach to the equilibrium with an increasing inlet flow rate, while that of q_o_ showed an opposite trend, showing that the EBCT decreased [5,7,47]. An increase in q_o_ with decreasing flow rates is the result of a longer interaction time between fluoride ions and adsorption sites [5,49,50]. Consequently, a lower flow rate results in a higher value of q_O_. The regression coefficient R^2^ being high (ranging from 0.976 to 0.996 for ZrOCSc, and from 0.953 to 0.994 for VSco), as displayed in Table 3, advocated that the Thomas model exhibited a good fit to the experimental adsorption data gained in the present work. The values obtained from the optimization of the Thomas model confirmed that the zirconium oxide coating improved the fluoride removal performance of VSco.

### 2.4. Application of the Adams–Bohart Model

**Figure 9:**
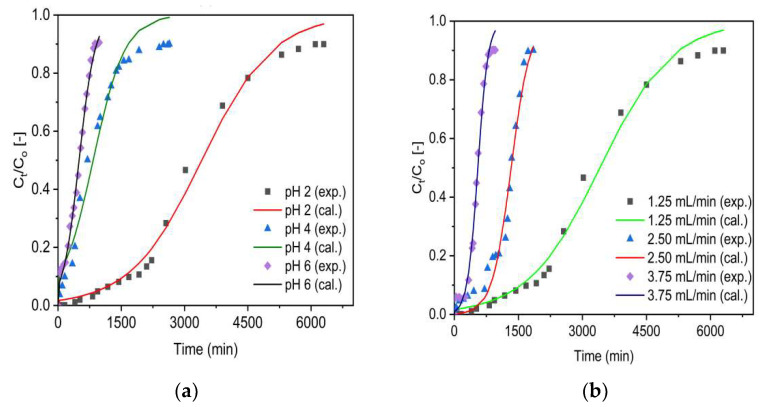
Experimental (exp.) and simulated (cal.; Adams–Bohart model) breakthrough curves of fluoride for ZrOCSc at different (**a**) pH (C_O_: 10 mg/L; Q_O_: 1.25 mL/min; bed depth 10 cm) and (**b**) Q_O_ (pH 2; C_O_: 10 mg/L; bed depth 10 cm).

**Figure 10:**
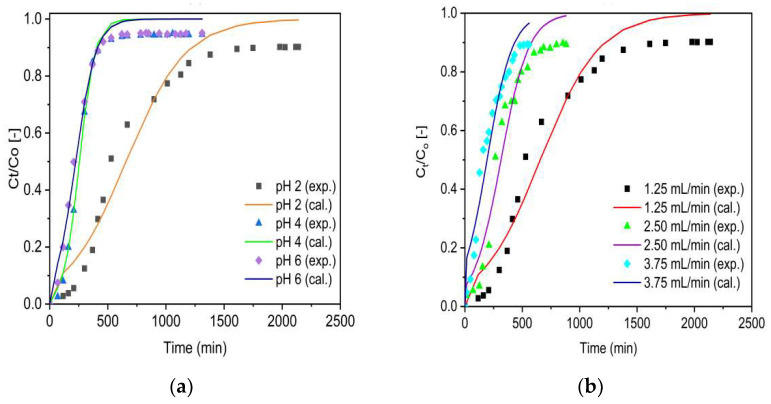
Experimental (exp.) and simulated (cal.; Adams–Bohart model) breakthrough curves of fluoride for VSco at different (**a**) pH (C_O_: 10 mg/L; Q_O_: 1.25 mL/min; bed depth 10 cm) and (**b**) Q_O_ (pH 2; C_O_: 10 mg/L; bed depth 10 cm).

Plots for experimental (denoted as exp.) and simulated (denoted as cal.) breakthrough data based on the Adams–Bohart model (Equation (13)) for VSco and ZrOCSc at various initial pH values and initial flow rates are shown in Figure 9 and Figure 10, respectively. 

As can be seen from Figure 9 and Figure 10, the breakthrough curves looked to move from right to the left as the pH and inlet flow rate increased from 2 to 6 and 1.25 to 3.75 mL/min, respectively. The Adams–Bohart rate constant (K_AB_) and adsorption capacity of the adsorbents per unit volume of the bed (N_O_) predicted by the model are presented in Table 4. Similar to the Thomas model, as the initial flow rate increased from 1.25 to 3.75 mL/min, the maximum volumetric adsorptive capacity of the bed (N_O_) decreased from 83 to 40 mg/L for ZrOCSc and 16 to 14 mg/L for VSco. Furthermore, the values of kinetics constant K_AB_ increased and values of EBCT decreased as the flow rate increased. This resulted in decreased N_O_ values. A decrease in N_O_ values with increasing flow rates is ascribed to the reduction in EBCT due to the direct proportion of the adsorption capacity to the interaction time. This is in agreement with previous studies [5,7,50]. As a result, the higher flow rate leads to a lower N_O_ value due to shorter interaction between the adsorbents and fluoride ions. The values of N_O_ decreased from 83 to 12 (mg/L) for ZrCOSc and from 16 to 5 (mg/L) for VSco with increased pH, while the other conditions remained constant (Table 4). The value of the coefficient of determination (R^2^) varied from 0.976 to 0.996 for ZrOCSc, and from 0.953 to 0.993 for VSco. The high values of R^2^ indicate the goodness of fit between the experimental data and the corresponding simulated data by the model. Thus, the model is suitable for depicting the adsorption behavior of fluoride in ZrOCSc and VSco. The results of the Adams–Bohart model optimization confirmed that the zirconium oxide coating improved the defluoridation performance of VSco.

On whole, the disparity between the Thomas and Adams–Bohart parameters for fluoride uptake onto ZrOCSc and VSco at different experimental parameters (as depicted in Table 3 and Table 4) indicated that ZrOCSc performed better than VSco in defluoridation. This proved that the zirconium oxide coating positively affected the defluoridation potential of VSco.

### 2.5. Fluoride Adsorption Performance of Different Adsorbents

Table 5 compares the adsorbent (ZrOCSc) used in this study with previously studied adsorbents for fluoride uptake in a flow-through fixed-bed column system.

Table 5 shows that the fluoride uptake capacity of ZrOCSc used in this study is greater than that of VSco* and VSco**. Furthermore, as shown in Table 5, ZrOCSc with a short bed height and a high initial fluoride concentration could be safely comparable with the adsorption performance of granular acid-treated bentonite and aluminum-modified iron, both of which have relatively long bed heights and low initial concentrations. Above all, unlike some of the other substrates, the raw material (VSco) is easily accessible and readily available, confirming that ZrOCSc could be a viable option for fluoride uptake from water. However, further investigation to improve its adsorption capacity might be considered.

## 3. Materials and Methods

### 3.1. Adsorbent Preparations

#### Coating of Zirconium Oxide onto Virgin Scoria (VSco)

**Figure 11:**
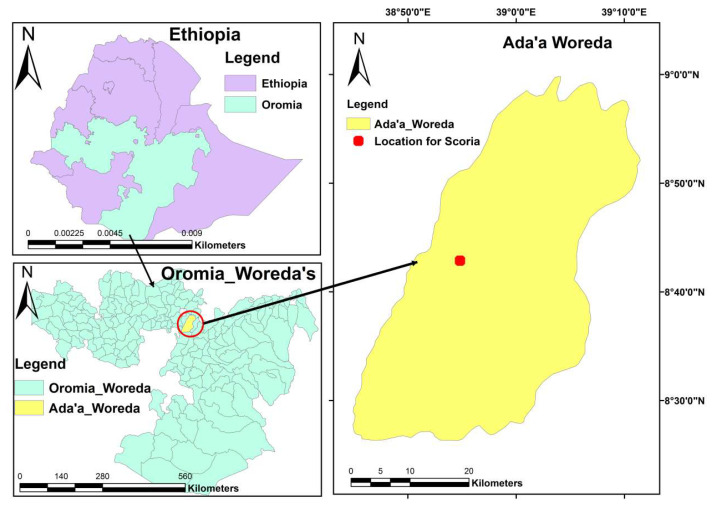
Sampling site of VSco.

The virgin scoria (VSco) used as a base material in zirconium oxide coating for surface modification was collected from volcanic cones (Figure 11) of the Main Rift Valley of Ethiopia; roughly 50 to 100 km East of Addis Ababa. The sample was washed multiple times with deionized water before being dried at 70 °C for 48 h. After cooling to room temperature, the sample was crushed and sieved to four different size fractions, as shown in [7]. In our previous study [7], virgin scoria (VSco) with a silt size (<0.075 mm) showed a good fluoride removal performance compared with the remaining three particle sizes. However, some studied and reported defluoridation materials are either fine particles or powder, which may make separation from an aqueous solution difficult. When used in fixed-bed adsorption systems, such materials could also cause clogging and/or low hydraulic conductivities [30]. To overcome these limitations, a fraction range of 0.075–0.425 mm was used for coating with zirconium oxide.

VSco coating was accomplished by completely soaking the dried sample in a sufficient amount of 0.1 M ZrOCl_2_·8H_2_O in acid-washed cylindrical polyethylene wide-mouth plastic bottles. The mixture was shaken with a horizontal shaker (SM25, Edmund Bühler 7400 Tübinger, Germany) at 200 rpm for 12 h. The zirconium oxide-coated scoria (ZrOCSc) was decanted, dried in an electric oven at 70 °C for 48 h, and soaked in 2 M NH_4_OH. The ZrOCSc was washed repeatedly with deionized water and dried at 70 °C for 48 h [5,53]. The coated VSco (ZrOCSc) was then packed and stored in an airtight plastic bag for use.

### 3.2. Chemicals and Reagents

All chemicals and reagents used in the experiments were of analytical grade. Zirconium oxychloride (IV) octahydrate (ZrOCl_2_·8H_2_O), ammonium hydroxide (NH_4_OH), sodium hydroxide (NaOH), and hydrochloric acid (HCl) were acquired from Merck KGaA, Darmstadt, Germany. An amount of 1000 mg/L of fluoride stock solution was gained by dissolving 2.21 g of NaF in 1000 mL of deionized water. The adsorbate solution concentration needed for the fixed-bed adsorption experiments was obtained by subsequent dilution of the stock solution with deionized water. The adsorbate solutions’ pH values were calibrated with 0.1 M NaOH and/or 0.1 M HCl. 

### 3.3. Characterizations of the Materials

The X-ray diffraction (XRD) patterns of ZrOCSc and VSco were gained by the XRD instrument (XRD-7000, Drawell, Shanghai, China) with Cu Kα as a radiation X-ray source (1.54056 Å) generated at 30 kV and 25 mA instrument. The diffractogram was achieved with a step width of 2θ in the range between 10° to 70° and a scan rate of 0.01°/min. The mineralogy content of the adsorbents was characterized by matching the diffractogram of VSco before adsorption and ZrOCSc before and after adsorption with the database of the X’pert HighScore Plus software package (Malvern Panalytical, Worcester, UK, 2007, Version: 2.2b (2.2.2)).

The oxide and elemental composition of the adsorbents were obtained by X-ray fluorescence (XRF, Mini-Pal 2 spectrometer, Panalytical, Malvern, Worcestershire, UK)) spectroscopy and inductively coupled plasma-optical emission spectroscopy (ICP-OES, Varian Vista MPX, Palo Alto, CA, USA), respectively. 

The Fourier-transform infrared (FTIR) spectra were recorded in a PerkinElmer spectrometer (UNSW, Sydney, Australia) over a range of 5000 to 400 cm^−1^ at a resolution of 0.1 cm^−1^ using a lithium tantalite (LiTaO_3_) detector.

The pH of the adsorbents was determined using a pH meter using a 1:10 adsorbent/water ratio according to the standard method. The point of zero charges (pH_PZC_) of the adsorbents was determined using 0.01 M of NaCl solutions as an electrolyte and adding 0.1 M of NaOH or HCl solutions for pH adjustment [5,7].

The scanning electron microscope (SEM) images were gained by NeoScope JCM-6000plus, Version 0.2, JEOL Ltd., Peabody, MA, USA, operated at 15 kV.

Simultaneous thermogravimetric and differential thermal analysis (TGA/DTA) of VSco and ZrOCSc were carried out using DTG-60H, SHIMADZU Corporation, Kyoto, Japan. An initial mass (about 12 ± 0.5 mg) was placed in an aluminum crucible at a heating rate of 15 °C/min from 25 to 900 °C under nitrogen purging (50 mL/min).

### 3.4. Fixed-Bed Column Adsorption Studies

Fixed-bed column tests of VSco and ZrOCSc were conducted in a small-scale cylindrical filter column (8.1 cm in diameter and 10 cm in height), as indicated in [5,7]. A weighted amount of material was packed carefully into the column. The bed was conditioned with one pore volume of deionized water to ensure a compact adsorbent [5]. An adjustable variable flow peristaltic pump (REGLO ICC, Ismatec, Cole-Parmer Barrington, IL, USA) was utilized to set the flow rate. All column tests were performed at 298 K. Column effluent samples were collected regularly by the automatic fraction collector (RFI, MA-RON GmbH, Reichelt Chemietechnik GmbH + Co., Heidelberg, Germany). The effluent samples’ fluoride concentration was measured by ion chromatography (930 Compact IC Flex, Metrohm, Herisau, Switzerland). The WHO standard for the fluoride content in drinking water (<1.5 mg/L) [54] was considered a breakthrough concentration (C_b_). The bed exhaustion/saturation point was considered when the fluoride level in the effluent was equal to 90% of the fluoride level in the influent (i.e., 0.9 C_t_/C_O_).

The influence of experimental parameters such as influent solution pH (2, 4, and 6) and influent volumetric flow rate (1.25, 2.50, and 3.75 mL/min) on the shape of the breakthrough curves and the amount of fluoride removed by the adsorbents was investigated at constant bed height (10 cm) and initial fluoride concentration (10 mg/L). 

### 3.5. Analysis of Column Data

#### Breakthrough Curve

A breakthrough curve is used to assess the dynamic adsorption process of a system and to predict the performance of the fixed-bed column system [55]. The breakthrough time and breakthrough curve pattern are important indicators for operational adsorption techniques. The viability and economics of the adsorption process are primarily related to these two parameters [6,7,56]. The experimental conditions such as influent pH and influent flow rate influence the profile of the breakthrough curve and its parameters. To study the performance and scaling of the fixed bed column, it is very imperative to study these parameters through experimental tests. The breakthrough curve was expressed by plotting (C_t_/C_O_) versus contact time, t, where C_O_ and C_t_ are the initial and the effluent fluoride concentration, respectively.

The time for exhaustion and the time for a breakthrough are given by the following Equations (3) and (4), respectively.

(3) $$t_{e}=\int_{t=0}^{t=t_{\mathrm{total}}}\left(1-\frac{C_{t}}{C_{o}}\right)\mathrm{dt}$$

(4) $$t_{b}=\int_{t=0}^{t_{b}}\left(1-\frac{C_{\mathrm{bt}}}{C_{o}}\right)\mathrm{dt}$$

where t_e_ is exhaustion time (min), and t_b_ is the breakthrough time (min) at which C_t_ = C_b_ (mg/L) (for the present system, C_b_ = 1.5 mg/L).

The total amount of fluoride adsorbed in a fixed-bed column, q_total_ (mg), was evaluated from the area (A) under the breakthrough curve using Equation (5).

(5) $$q_{\mathrm{total}}=\frac{\mathrm{QA}}{1000}=\frac{Q\times C_{O}}{1000}\int_{t=0}^{t=t_{\mathrm{total}}}\left(1-\frac{C_{t}}{C_{O}}\right)\mathrm{dt}$$

where t_total_ and Q are the total flow time until saturation of the bed (min) and flow rate (mL/min), respectively.

The maximum fluoride removal capacity (q_e_: mg kg^−1^) of the column was calculated using Equation (6).

(6) $$q_{\mathrm{eq}}=\frac{q_{\mathrm{total}}}{m}=\frac{C_{o}{\mathrm{Qt}}_{e}}{m}$$

The amount of fluoride removed at t_b_ (q_b_: mg kg^−1^) can be determined by Equation (7).

(7) $$q_{b}=\frac{C_{o}{\mathrm{Qt}}_{b}}{m}$$

The effluent volume (V_e_) and treated effluent volume or breakthrough volume (V_b_) can be evaluated with Equations (8) and (9), respectively.

(8) $$V_{e}={\mathrm{Qt}}_{e}$$

(9) $$V_{b}={\mathrm{Qt}}_{b}$$

where V_e_ is the total effluent volume at saturation time (mL), and V_b_ is the total effluent volume at the breakthrough time (mL).

The mass transfer zone (MTZ) or unused bed length (H_UNB_) can be evaluated from Equation (10).

(10) $$\mathrm{MTZ}=H_{T}\left(\frac{t_{e}-t_{b}}{t_{e}}\right)$$

where H_T_ is total bed height (cm), t_e_ (min) is exhaustion time, and t_b_ is breakthrough time (min).

The empty bed contact time (EBCT), which is defined as the contact time between the solid phase adsorbent and the liquid phase, can be determined from Equation (11).

(11) $$\mathrm{EBCT}=\frac{V_{B}}{Q}$$

where V_B_ is the volume of a fixed bed (mL), and Q is the flow rate (mL/min).

### 3.6. Breakthrough Curve Modeling

The modeling of breakthrough curves can efficaciously avoid extensive studies on the pilot scale [57]. Various models have been reported to estimate breakthrough performance in the fixed-bed adsorption process [12]. In this work, the two most common and widely employed mathematical models—the Thomas model and Adams–Bohart model—were used in the experimental data of the column to depict the dynamic behavior of fluoride uptake onto VSco and ZrOCSc packed fixed-bed column filter. The coefficient of determination (R^2^) was used to estimate the validity of the models. The mathematical descriptions of the models are given in the following sub-sections.

#### 3.6.1. Thomas Model

The Thomas model [58] is among the most prominent models in fixed-bed column studies to anticipate the maximum adsorption capacity (q_o_) and the adsorption rate constant (K_T_). The model was also used to forecast effluent breakthrough curves. The model is based on the following assumptions [59]: (i) the plug flow characteristic occurs in the fixed-bed; (ii) the external and internal diffusion constraints are insignificant; and (iii) the experimental data follow the Langmuir isotherm and second-order reversible reaction kinetics. The non-linear form of the Thomas model is given in Equation (12), as follows.

(12) $$\frac{C_{t}}{C_{o}}=\frac{1}{1+\exp\left[K_{T}q_{o}\frac{m}{Q}-K_{T}C_{o}t\right]}$$

where C_O_ (mg/L) is the initial solute concentration, C_t_ (mg/L) is the solute concentration at the time, t, Q (L/min) is the volumetric flow rate, q_o_ (mg/kg) is the adsorbed fluoride at equilibrium, K_T_ is the model kinetic constant (L/min mg), and m (kg) is the dry adsorbent mass.

#### 3.6.2. Adams–Bohart Model

The Adams–Bohart model [60] was developed for the analysis of the dynamics of fixed-bed under the assumption that the adsorption rate is not instantaneous and that the adsorption rate is proportional to the residual adsorption capacity of the adsorbent and the concentration of adsorbate. The non-linear form of the Adams–Bohart model (Equation (13)) [61] was used to estimate the breakthrough curves and the model parameters.

(13) $$\frac{C_{t}}{C_{o}}=\frac{1}{1+\exp\left[K_{\mathrm{AB}}N_{o}\frac{Z}{v}-K_{\mathrm{AB}}C_{o}t\right]}$$

where K_AB_ (L/mg min) is the kinetic constant of the model, *v* (mL/min) is the linear flow rate, Z (cm) is the depth of the column bed, and N_O_ (mg/L) is the saturation concentration (adsorption capacity of the adsorbent per unit volume of the bed), and time t (min). 

The linear flow rate was determined using Equation (14).

(14) $$v=\frac{Q}{A}$$

where Q (mL/min) is the volumetric flow rate, and A (cm^2^) is the cross-sectional area of the bed.

## 4. Conclusions

In this work, virgin scoria (VSco) and zirconium oxide-coated scoria (ZrOCSc) were examined for fluoride adsorption in fixed-bed column systems. The XRD analysis revealed that the VSco surface was coated with zirconium oxide. The absence of harmful substances on both adsorbents and the amount of zirconium oxide coated on VSco was evident from ICP-OES and XRF analysis. The FTIR analysis displayed an insignificant disparity between VSco and ZrOCSc spectra before and after fluoride adsorption. The recorded SEM image clearly showed the degree of surface modification with improved porosity. Thermal analysis of VSco and ZrOCSc showed lower overall weight losses of 2.3 and 3.2 percent, respectively, due to the removal of water molecules and OH species bound to metal oxides contained in the material. The pH_PZC_ analysis depicted that the surface charge of VSco and ZrOCSc was positive when the pH of the solution was below pH_PZC_ of 8.3 and 8.7, respectively. The defluoridation capability of the ZrOCSc was 4.46 times greater than that of VSco under optimum experimental conditions (pH 2 and influent flow rate of 1.25 mL/min). The breakthrough time of ZrOCSc was 6.66 times longer than that of VSco; consequently, the treated water volume at breakthrough for ZrOCSc was 2573 mL (6.66 times that of VSco). This improved performance could be ascribed to the zirconium oxide coating onto the VSco. The experimental results were well fitted by the Adams–Bohart and Thomas models, indicating that the attained models’ parameters could be used to upscale the design of ZrOCSc- and VSco-based defluoridation filters without the need for additional experimentation. According to this study, the coating of a low-cost adsorbent material, VSco, with zirconium oxide had a beneficial influence on its surface and improved its defluoridation performance. Therefore, ZrOCSc is a worthy material for eliminating high levels of fluoride in groundwater. However, further studies, such as regeneration and competing anions tests, are needed to conclude that the defluoridation of water with ZrOCSc is an economically viable and sustainable process.

## Figures and Tables

**Table 1:** Elemental and oxide compositions of virgin scoria (VSco) and zirconium oxide-coated scoria (ZrOCSc).

Elemental Content	VSco % (wt)	ZrOCSc % (wt)	Oxide Content	VSco % (wt)	ZrOCSc % (wt)
Si	18.3	19.3	SiO_2_	47.4	44.8
Al	10.3	10.2	Al_2_O_3_	21.6	23.1
Fe	7.8	8.1	Fe_2_O_3_	8.9	5.8
K	0.4	0.4	K_2_O	0.5	0.3
Ca	6.4	6.2	CaO	12.4	11.9
Na	2.2	2.2	Na_2_O	3.0	2.6
Mg	2.8	2.7	MgO	3.3	2.0
Zn	<0.1	<0.1	TiO_2_	1.4	1.1
Zr	<0.1	1.2	ZrO_2_	-	8.3
Mn	0.1	0.1	MnO	0.4	0.1
Cr	<0.1	<0.1	ZnO	0.2	0.2
Cu	<0.1	<0.1	NiO	0.1	0.2
Co	<0.1	<0.1	CuO	0.2	0.2
Cd	<0.1	<0.1	-	-	-
Ni	<0.1	<0.1	-	-	-
Pb	<0.1	<0.1	-	-	-
As	<0.1	<0.1	-	-	-

**Table 2:** Fixed-bed column parameters obtained for defluoridation by VSco and ZrOCSc.

Parameter Studied	pH	C_O_ (mg/L)	Q_O_ (mL/min)	EBCT (min)	t_b_ (min)	t_e_ (min)	V_b_ (mL)	V_e_ (mL)	MTZ (cm)	q_b_ (mg/kg)	q_tot_ (mg)	q_e_ (mg/kg)	Adsorbent
Variation in pH keeping C_O_ and Q_O_ constant	2	10	1.25	412	2058	3425	2572.50	4280.84	3.99	34.86	42.81	58	ZrOCSc
	4	10	1.25	412	316	944	394.26	1180.41	6.66	5.34	11.80	16	
	6	10	1.25	412	161	495	200.89	618.75	6.75	2.72	6.19	8.39	
	2	10	1.25	412	309	783	386.25	941.25	5.60	5.23	9.41	12.76	VSco
	4	10	1.25	412	137	318	170	386.25	5.70	2.30	3.86	5.23	
	6	10	1.25	412	91	275	114.13	343.75	6.68	1.55	3.44	4.66	
Variation in Q_O_ keeping pH and C_O_ constant	2	10	1.25	412	2058	3425	2572.50	4280.84	3.99	34.86	42.81	58	ZrOCSc
	2	10	2.50	206	721	1262	1803.05	3153.60	4.28	24.44	31.54	42.54	
	2	10	3.75	137	336.23	647.67	1260.86	2053.76	4.81	17.09	20.54	27.83	
	2	10	1.25	412	309	753	386.25	941.25	5.90	5.53	9. 51	12.76	VSco
	2	10	2.50	206	155	353	386.58	881.58	5.61	5.24	8.82	11.95	
	2	10	3.75	137	65	217	243.41	813.49	7.01	3.30	8.15	11.02	

t_b_ = breakthrough time, t_e_ = exhaustion time, V_b_ = total effluent volume at a breakthrough time, V_e_ = total effluent volume at exhaustion time MTZ = mass transfer zone, EBCT = empty bed contact time, q_b_ = amount of fluoride removed at a breakthrough time per kg of adsorbent, q_tot_ = total amount of fluoride adsorbed from the column, q_e_ = equilibrium fluoride uptake per kg of the adsorbent.

**Table 3:** Thomas model parameter values for the defluoridation by VSco and ZrOCSc.

Parameter Studied	pH	C_O_ (mg/L)	Q (mL/min)	Bed-Depth, H_B_ (cm)	K_T_ (L/min·mg) (×10^4^)	q_o(cal.)_ (mg/kg)	q_e(exp.)_ (mg/kg)	R^2^	Adsorbent
Variation in pH keeping C_O_, and Q_O_ constant	2	10	1.25	10	1.192	57.71	58	0.992	ZrOCSc
	4	10	1.25	10	2.642	14.13	16	0.976	
	6	10	1.25	10	5.253	8.45	8.39	0.996	
	2	10	1.25	10	3.892	11.12	12.76	0.967	VSco
	4	10	1.25	10	15.083	4.34	5.23	0.977	
	6	10	1.25	10	15.634	3.78	4.66	0.994	
Variation in Q_O_ keeping pH and C_O_ constant	2	10	1.25	10	1.192	57.71	58	0.992	ZrOCSc
	2	10	2.50	10	4.542	45.09	42.54	0.980	
	2	10	3.75	10	8.277	28.03	27.83	0.993	
	2	10	1.25	10	3.892	11.12	12.76	0.967	VSco
	2	10	2.50	10	8.333	10.66	11.95	0.961	
	2	10	3.75	10	9.127	9.81	11.02	0.953	

**Table 4:** Adams–Bohart model parameter values for defluoridation by ZrOCSc and VSco.

Parameter Studied	pH	C_O_ (mg/L)	Q (mL/min)	Bed-Depth, H_B_ (cm)	K_AB_ (L/min·mg) (×10^4^)	N_O(cal.)_ (mg/L)	R^2^	Adsorbent
Variation in pH keeping C_O_ and Q_O_ constant	2	10	1.25	10	1.192	82.78	0.992	ZrOCSc
	4	10	1.25	10	2.642	20.26	0.976	
	6	10	1.25	10	5.253	12.12	0.996	
	2	10	1.25	10	3.892	15.95	0.967	VSco
	4	10	1.25	10	15.083	6.23	0.977	
	6	10	1.25	10	15.634	5.43	0.994	
Variation in Q_O_ keeping pH and C_O_ constant	2	10	1.25	10	1.192	82.78	0.992	ZrOCSc
	2	10	2.50	10	4.542	64.55	0.980	
	2	10	3.75	10	8.277	40.16	0.993	
	2	10	1.25	10	3.892	15.95	0.967	VSco
	2	10	2.50	10	8.333	15.26	0.961	
	2	10	3.75	10	9.127	14.05	0.953	

**Table 5:** Fluoride uptake capacity of some reported adsorbents.

Adsorbents	Bed Height (cm)	Fluoride Level in (mg L^−1^)	Adsorption Capacity (mg g^−1^)	References
Granular acid-treated bentonite	28	6.34	0.190	[51]
Granular acid-treated bentonite	28	2.85	0.169	[51]
Aluminum-modified iron oxide	10.5	4	0.139	[52]
Virgin Scoria (VSco*)	10	10	0.022	[7]
Virgin Scoria (VSco**)	10	10	0.013	[7]
ZrOCSc	10	10	0.058	This study

VSco*: <0.075 mm; VSco**: 0.075–0.425 mm.

## Data Availability

The data used in this study can be available from the authors at reasonable request.

## Sample Availability

Samples/adsorbents used in this study are available from the authors based on reasonable request.
